# Flavonols in Action: Targeting Oxidative Stress and Neuroinflammation in Major Depressive Disorder

**DOI:** 10.3390/ijms24086888

**Published:** 2023-04-07

**Authors:** Maja Jazvinšćak Jembrek, Nada Oršolić, Dalibor Karlović, Vjekoslav Peitl

**Affiliations:** 1 Division of Molecular Medicine, Ruđer Bošković Institute, Bijenička 54, 10000 Zagreb, Croatia; 2 School of Medicine, Catholic University of Croatia, Ilica 242, 10000 Zagreb, Croatia; 3 Division of Animal Physiology, Faculty of Science, University of Zagreb, Rooseveltov trg 6, 10000 Zagreb, Croatia; 4 Department of Psychiatry, Sestre Milosrdnice University Hospital Center, 10000 Zagreb, Croatia

**Keywords:** depression, flavonols, neuroinflammation, oxidative stress, HPA axis

## Abstract

Major depressive disorder is one of the most common mental illnesses that highly impairs quality of life. Pharmacological interventions are mainly focused on altered monoamine neurotransmission, which is considered the primary event underlying the disease’s etiology. However, many other neuropathological mechanisms that contribute to the disease’s progression and clinical symptoms have been identified. These include oxidative stress, neuroinflammation, hippocampal atrophy, reduced synaptic plasticity and neurogenesis, the depletion of neurotrophic factors, and the dysfunction of the hypothalamic–pituitary–adrenal (HPA) axis. Current therapeutic options are often unsatisfactory and associated with adverse effects. This review highlights the most relevant findings concerning the role of flavonols, a ubiquitous class of flavonoids in the human diet, as potential antidepressant agents. In general, flavonols are considered to be both an effective and safe therapeutic option in the management of depression, which is largely based on their prominent antioxidative and anti-inflammatory effects. Moreover, preclinical studies have provided evidence that they are capable of restoring the neuroendocrine control of the HPA axis, promoting neurogenesis, and alleviating depressive-like behavior. Although these findings are promising, they are still far from being implemented in clinical practice. Hence, further studies are needed to more comprehensively evaluate the potential of flavonols with respect to the improvement of clinical signs of depression.

## 1. Introduction

Major depressive disorder is a severe neuropsychiatric disease driven by various hereditary, environmental, and psychological factors [1]. It disables normal functioning and quality of life. Typical symptoms include ongoing sadness, feelings of guilt, fatigue, loss of energy, lack of motivation for engaging in previously rewarding activities, impairment of cognitive functions, and disturbances of sleep, weight, and appetite. Depression is a common mental disorder and is one of the leading causes of disability worldwide. Globally, more than 350 million people suffer from depression, with the prevalence being higher among women [2,3].

The clinical manifestations of depression are accompanied by morphological changes of specific brain regions mainly related to mood regulation, rewards, emotions, and cognitive functions (such as decision making and memory). Neuroimaging studies have revealed significant structural alterations, together with abnormal interregional connectivity and brain activity, in various cortical and limbic brain areas. Affected areas include the frontal lobe (e.g., the orbitofrontal and dorsolateral prefrontal cortexes), the hippocampus, the thalamus, the basal ganglia, the amygdala, and the anterior cingulate cortex as well as cerebellar neurons projecting toward the ventral tegmental area (VTA) [4,5,6,7,8,9]. However, due to the diversity of demographic and clinical data, methodologies used, and study designs, the results concerning the identification of the depression-related patterns of brain changes are very heterogeneous and far from generalization.

## 2. Neuropathological Mechanisms Underlying the Development of Depression

### 2.1. Monoamine Hypothesis of Depression

The mechanisms underlying the etiology of depression are still not fully understood. From a clinical perspective, the typical abnormality witnessed is a disruption of neurotransmitter levels, particularly those of serotonin, dopamine, and norepinephrine. Depressed patients have reduced levels of monoamines and their metabolites in the cerebrospinal fluid and the post-mortem brain, although the results on this matter are not completely uniform and conclusive [10,11,12,13]. The major mechanisms related to monoamine depletion include the impaired activity of enzymes participating in their degradation, the impaired activity of enzymes involved in tryptophan synthesis, and the impaired activity of monoamine transporters and receptors [14,15,16]. Moreover, most of the behavioral symptoms of depressed patients can be related to disruptions in monoamine levels [17]. Hence, the monoamine hypothesis contends that monoamine deficiency is the core mechanism underlying the pathophysiological events of depression [18]. This hypothesis is supported by the mechanisms of action of clinically used antidepressants. The main pharmacological approaches are based on improving the monoamine balance by using selective serotonin reuptake inhibitors (SSRIs); tricyclic antidepressants that inhibit the reuptake of serotonin, dopamine, and norepinephrine; monoamine oxidase inhibitors; and selective norepinephrine reuptake inhibitors (NARI) [19,20]. The first-line drugs employed are usually SSPIs such as fluoxetine, sertraline, and citalopram, which slowly increase synaptic serotonin levels. However, despite this repertoire of available drugs, many patients do not respond adequately to such therapy [21], and many unwanted side effects are induced alongside the beneficial ones. Some of the adverse effects reported by patients include dizziness, sedation, anxiety, cardiac problems, gastrointestinal and sexual disfunction, sleep disturbances, appetite changes, forgetfulness, confusion, difficulty finding words, and other cognitive impairments [22].

Besides the limited efficacy and slow onset of the most-prescribed drugs, monoamine depletion neither causes depression in healthy individuals nor worsens symptoms in patients not undergoing therapy, thereby raising questions concerning the exact role of monoamine deficiency in the etiopathology of depression and suggesting the involvement of other pathological mechanisms [18,23]. More recent studies have demonstrated neurochemical, structural, and functional impairments of GABAergic and glutamatergic systems. As will be explained below, these dysfunctions are likely mediated by excitotoxicity and increased levels of pro-inflammatory cytokines and adrenal glucocorticoids in combination with certain environmental factors [24]. Evidence revealing that the impaired function of GABAergic and glutamatergic systems is implicated in the pathophysiology of depression resulted in advancements in therapeutic strategies, particularly with respect to the treatment-resistant depression, leading to the introduction of fast-acting antidepressants targeting N-methyl-D-aspartate (NMDA) and γ-aminobutyric acid type A (GABA_A_) receptors [24,25,26].

### 2.2. Hippocampus and Depression

Brain imaging has revealed lesions mainly localized in the prefrontal and cingulate cortex, hippocampus, and amygdala [8,27], indicating increased susceptibility to neuronal death in distinct brain areas. Structural and functional alterations of the hippocampus are particularly relevant considering this structure’s role in the regulation of stress response, memory-related consolidation processes, and adult neurogenesis. Depressed patients have reduced hippocampal volume due to a decreased number of both neuronal and glial cells, suggesting that hippocampal changes are likely a contributing factor in the pathophysiology of depression [4,28,29]. At the cellular and molecular level, it is believed that the dysregulation of the HPA axis and the consequent increase in the quantity of glucocorticoids are the underlying mechanisms of reduced neurogenesis, excitotoxicity, inflammation, and depleted levels of neurotrophins, which all contribute to reducing the size of the hippocampus [4].

The severity of hippocampal degeneration positively correlates with the duration of the symptoms and negatively correlates with the efficacy of antidepressant therapies and clinical outcomes [30,31,32]. Boku et al. proposed two hypotheses for explaining changes in the hippocampal volume [33]. The neuroplasticity hypothesis emphasizes the degenerative changes of mature neurons in the hippocampus (the reduced branching and length of dendrites and the reduced density of dendritic spines), whereas the neurogenesis hypothesis considers the reduced neurogenesis and the role of the neural progenitor cells in the dentate gyrus. These hippocampal deteriorations are considered to be largely responsible for delayed responses to antidepressant therapy. It has been shown that only long-term treatment with antidepressants protects mature neurons and promotes the proliferation of neural progenitors and neurogenesis. At least partially, the protection of mature neurons is mediated through increased 5-HT_4_-receptor-mediated signaling [34], whereas neurogenesis is stimulated by glucocorticoid-receptor-initiated pathways [35]. Thus, the time needed to observe effects on hippocampal neurons corresponds with the therapeutic window required to observe the improvement of clinical manifestations [28,29].

#### Adult Neurogenesis and Depression

The process of neurogenesis is tightly related to neurotrophic factors. Brain-derived neurotrophic factor (BDNF) belongs to the neurotrophin family and is one of the most abundant neurotrophins in the brain. BDNF acts via the tropomyosin receptor kinase B (TrkB) receptor, which is expressed on both neuros and glia. The activation of TrkB receptors triggers the activation of signaling cascades involved in neuronal survival and functioning, including the mitogen-activated protein kinase (MAPK), phosphatidylinositol 3-kinase (PI3K)/Akt, and phospholipase C-γ (PLC-γ) pathways [36,37]. BDNF plays an important role in the wide spectrum of neurophysiological processes. Among other functions, it regulates neuronal development and survival, synaptogenesis, plasticity, and learning and memory [38,39,40,41]. Different BDNF roles are mediated by distinct transcripts that arise from the nine promotors in the *BDNF* gene, which all encode the same BDNF protein [42]. These transcripts are expressed in a complex spatio-temporal pattern, respond to different stimuli, and trigger the activation of distinct signaling networks [43,44]. Hence, depending on the brain region, the effects of BDNF could be antidepressant or pro-depressant. In the hippocampus and prefrontal cortex, BDNF displays antidepressant activity, and depressed patients usually exhibit reduced serum BDNF levels and levels of BDNF IV transcript in the hippocampus and prefrontal cortex [45,46,47]. The disruption of murine promotor IV impairs GABAergic transmission and the expression of monoaminergic genes in the hippocampus and prefrontal cortex and results in depression-like behavior [48,49,50]. Moreover, there are indications that the methylation of promotor IV and plasma BDNF levels can be used as a potential predictor of treatment response [51]. It has been proposed that serum BDNF concentrations may serve as the peripheral biomarker of the disease [45], yet its levels in the serum do not always correlate with the severity of symptoms [52,53,54]. However, BDNF concentrations increase after antidepressant therapy, and there is a good correlation between BDNF changes and improvements in depression scores [55,56].

### 2.3. HPA Axis and Depression

HPA hyperactivity is commonly observed in patients with chronic stress and depression [57,58,59]. The hippocampus possesses many receptors for glucocorticoids. Through a negative feedback mechanism, it regulates glucocorticoid release from the adrenal cortex, thus playing a critical role in the tuning of the HPA axis [60,61]. The HPA axis starts with the neurosecretory cells of the hypothalamus that release corticotrophin-releasing hormone (CRH). CRH stimulates the release of the adrenocorticotropic hormone (ACTH) from the anterior pituitary, whereas ACTH further stimulates the adrenal gland so that it both produces and releases glucocorticoids. In turn, through inhibitory feedback, the released glucocorticoids attenuate the production of CRH and ACTH via glucocorticoid and mineralocorticoid receptors. These receptors are particularly abundant in the hippocampus. The excitatory output from hippocampal neurons along with the activity of inhibitory GABAergic cells regulate CRF-releasing neurons in the hypothalamus. Under stressful conditions, the HPA axis escapes this regulatory mechanism and produces large amounts of glucocorticoids [60,62]. A long-term increase in glucocorticoid levels reduces dendritic branching and dendritic length and induces the death of mature neurons and progenitor cells, resulting in reduced hippocampal volume. Thus, the disruption of neuroendocrine regulation ultimately impairs the excitability, functions, and integrity of the hippocampus; reduces plasticity and neurogenesis; and promotes susceptibility to the development of depression [58,63,64,65,66,67,68,69].

### 2.4. Oxidative Stress and Depression

Depression is accompanied by increased oxidative stress and reduced concentrations of antioxidants in the plasma [70,71].

Briefly, oxidative stress refers to a condition in which there is an imbalance between the generation of reactive oxygen and nitrogen species (ROS/RNS) and the ability of various enzymatic and non-enzymatic mechanisms of endogenous defense to maintain their levels in a physiological range. In appropriate concentrations, ROS have important functions, acting as signaling molecules in various redox-sensitive signaling pathways. However, if present in excess, they disturb neuronal signaling and react with cellular lipids, proteins, and nucleic acids, thus threatening their structure and proper neuronal functioning [72,73,74,75]. The brain is particularly vulnerable to oxidative injury due to its high metabolic activity, high content of redox-active transition metals that act as initiators of ROS generation via Fenton chemistry, high content of poly-unsaturated fatty acids (PUFAs) that are highly prone to lipid peroxidation, and limited mechanisms of antioxidative defense, among other reasons [76].

**Figure 1:**
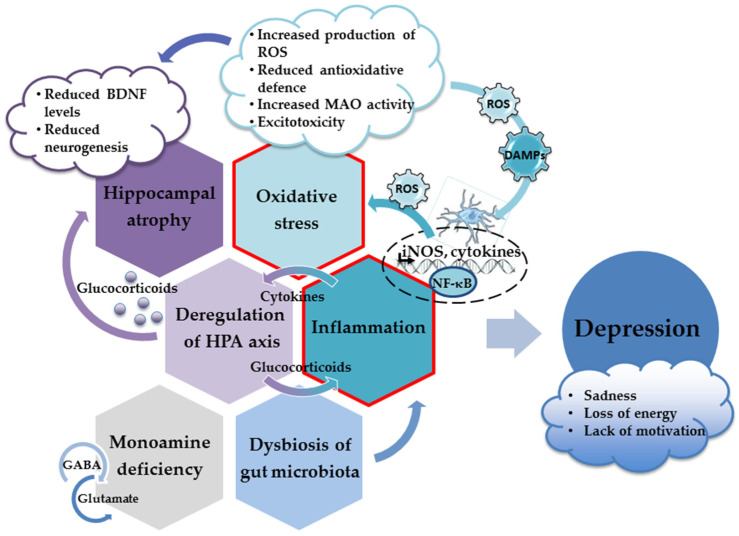
The main neuropathological mechanisms involved in the development of depression.

More importantly, specific molecules released or exposed during oxidative injury may elicit an innate immune response in the brain (acting as danger-associated molecular patterns (DAMPS)) and trigger sterile inflammation [77,78]. Thus, oxidative stress contributes to the inflammatory response and the increased production of proinflammatory cytokines, which inevitably results in the appearance of depressive behavior (Figure 1). Hence, both oxidative stress and neuroinflammation have been recognized as underlying factors in the development of depression [77,79,80,81].

The presence of oxidative stress markers is a common finding in depressed patients and animal models of depression [82]. 8-hydroxy-2′-deoxyguanosine (8-OHdG) is a typical indicator of oxidative DNA damage and it levels are significantly increased in blood samples from depressed patients [83,84]. The extent of lipid peroxidation in the peripheral blood is also increased and correlates with the severity of depressive symptoms [85,86,87,88]. Malondialdehyde (MDA), an end marker of lipid peroxidation, is usually found in increased concentrations in depressed patients [89]. Moreover, it has been suggested that certain oxidative stress indicators may be used as prognostic markers of disease severity and for the evaluation of the efficacy of administered antidepressants [82].

An increase in oxidative stress parameters reduces the content of intracellular mechanisms of antioxidative defense and jeopardizes neuronal protection. Thus, changes in the expression and activity of antioxidative and prooxidative enzymes have been observed; however, the results are not straightforward. Levels of the prooxidative enzyme xanthine oxidase, which generates superoxide anions and hydrogen peroxide, are usually increased in both depressed patients and animal models of depression [90,91]. On the other hand, levels of superoxide dismutase (SOD), an enzyme that decomposes superoxide to oxygen and hydrogen peroxide, were found to be reduced, unchanged, or enhanced in depressed patients [92,93,94,95]. Hydrogen peroxide is removed by catalase. Similarly, increased, unchanged, and decreased catalase activity has been reported [89,93,95]. Glutathione peroxidase also reduces hydrogen peroxide and regenerates reduced glutathione (GSH) from its oxidized pool (GSSG), thereby increasing the overall scavenging ability of GSH. The activity of glutathione peroxidase is predominantly found to be reduced in depressed patients, which is negatively correlated with the severity of the symptoms [93,96]. However, some studies did not observe changes in the glutathione peroxidase activity of depressed patients [84,92]. In yet another study, increases in the levels of glutathione peroxidase and SOD were dependent on the sample analyzed (plasma or erythrocytes) and clinical manifestations (depression with or without melancholia), suggesting that various factors affect the extent of changes in the activity of antioxidant enzymes in depressed patients [97]. Of note, the polymorphism of several genes participating in ROS metabolism may represent a risk for disease development and progression. It has been shown that single-nucleotide polymorphisms (SNPs) in genes encoding mitochondrial SOD (SOD2), catalase, and glutathione peroxidase 4 may modulate the risk of the onset of disease [98,99].

Quantities of non-enzymatic antioxidants are also disturbed. Besides developing oxidative stress that decreases the levels of these antioxidant molecules, depressed people often change their food preferences, which may deplete concentrations of specific dietary products, including vitamins [100]. Levels of vitamins A, C, and E as well as other small antioxidants (such as uric acid, albumin, and coenzyme Q10) are lower in depressed patients and negatively correlate with the disease’s severity [86,101,102,103]. Hence, various dietary approaches, such as supplementation with the bioactive antioxidative ingredients from certain types of food, have been considered as adjuvant strategies for mitigating depressive symptoms [104,105,106]. Several studies have shown that supplementation with vitamin C may induce antidepressant effects and improve moods [107,108]. However, in combination with citalopram therapy, vitamin C was as effective as a placebo [109], whereas in combination with fluoxetine, it significantly mitigated depressive symptoms in a pediatric population [110]. Similarly, results on supplementation with vitamin E are inconclusive and further studies are needed to clarify the possible benefits of vitamin E with respect to the management of depression [102,111,112]. Administration of N-acetyl cysteine, a GSH precursor, also provided promising evidence regarding its use as an adjuvant therapy for depression. Several studies have indicated its ability to reduce the severity of depression, improve moods, and increase the efficacy of standard antidepressant therapy, but these findings need further confirmation [113,114,115].

Metabolic reactions in the mitochondria are the major sources of ROS, but mitochondria are also the major targets of ROS action. Considering the principal role of mitochondria in determining cell death and survival via ATP production and the initiation of apoptosis, the preservation of mitochondrial functions is an important prerequisite for neuronal health [116,117]. In depressed patients, the expression and activity of enzymes participating in the mitochondrial respiratory chain are disrupted, leading to a reduction in ATP production [118,119]. A growing body of evidence is indicating that depression is related to mitochondrial dysfunction, which, consequently, disturbs ROS balance and promotes oxidative stress [120,121]. Depressed patients also have increased levels of circulating cell-free mtDNA (ccf-mtDNA), which likely reflects a fraction of the mitochondrial DNA released under cellular stress conditions [122].

Monoamine oxidase MAO-A and MAO-B, isoenzymes located in the outer mitochondrial membrane, catalyze the oxidative deamination of monoamines and deplete their levels in the brain, while also contributing to ROS production and oxidative stress. The MAO-A isoform is mainly involved in the metabolism of serotonin, dopamine, and norepinephrine, whereas MAO-B predominantly metabolizes dopamine. Increased activity of MAO-A is a common finding in depressed patients. It has been suggested that this upregulation is the predominant mechanism underlying monoamine loss [123,124,125]. Although MAO inhibitors were the first class of antidepressants developed and were used therapeutically for decades, based on concerns related to safety, possible side effects related to potential drug interactions, and dietary restrictions, they have been replaced with safer and more tolerable options. However, inhibitors of MAO activity are still considered the most valuable pharmacological option, particularly with respect to patients who fail to respond to first-line therapy [126,127,128].

Oxidative stress is usually accompanied by excitotoxicity, which is induced by the hyperactivation of glutamatergic NMDA receptors. It has been proposed that the impaired clearance and increased release of glutamate by activated glial cells upregulates glutamate levels and disturbs signaling through ionotropic and metabotropic glutamate receptors, thus contributing to neuronal dysfunction and, ultimately, behavioral changes [129]. Excitotoxicity also increases the production of NO, which, together with the superoxide anion, generates extremely cytotoxic peroxynitrite [130,131]. In addition, it has been shown that peroxynitrite inactivates tryptophan hydroxylase, the rate-limiting enzyme in serotonin synthesis [132]. Moreover, NO reacts with amino acid residues in proteins (mainly cysteine, tyrosine, tryptophan, and arginine) and causes protein oxidation, nitration, and nitrosylation, further threatening neuronal functioning [78,103,133]. However, peripheral measurements of nitrosative stress markers have provided variable results. For example, reduced levels of nitrate, without changes in NO and nitrite levels, have been reported in depressed patients [134].

There is yet another connection between depression and the activation of NMDA receptors. In inflammatory conditions, the kynurenine pathway and the production of kynurenine from tryptophan are potentiated, which per se reduce serotonin concentrations. On the other hand, kynurenine is metabolized by microglial enzymes, resulting in the production of various metabolites, some of which are neurotoxic, such as quinolinic acid, an NMDA receptor agonist [135,136].

Finally, the reduced expression of BDNF precipitates higher susceptibility to stress-induced oxidative damage, indicating an interplay between these depression-related parameters [137]. In turn, increased oxidative stress affects BDNF production and contributes to reduced neurogenesis and the dysregulation of hippocampal functioning [82].

### 2.5. Neuroinflammation, Cytokines, and Depression

Animal studies have provided evidence for a neuroinflammatory basis of depression, which entails sterile inflammation, glial activation, and the release of pro-inflammatory cytokines [138]. Depression is accompanied by a chronic, low-grade inflammatory state; the dysregulation of the innate and adaptive immune system; enhanced production of various cytokines, such as interleukin (IL)-1, IL-2, IL-6, IL-10, IL-12, IL-13, and IL-1β and tumor necrosis factor (TNF)-α; and decreased concentrations of interferon (INF)-γ [139,140,141,142,143]. Treatment with antidepressants reduces the severity of inflammation, while severe inflammation correlates with lower treatment efficacy [144,145].

The excessive production of cytokines, the signaling molecules of the immune system, affects neurocircuitry in the basal ganglia and anterior cingulate cortex, the functioning of the HPA axis, synaptic plasticity, and neurotransmission, leading to behavioral alterations, mood changes, and the impairment of cognitive functions characteristic of depression. The behavioral effects of cytokines are largely mediated through the activation of the inflammatory signaling pathways that regulate the production of monoamines, glutamate, neuropeptides, and BDNF [146,147,148,149].

An increase in glucocorticoid concentrations mediates the effects of cytokines on the activation of the HPA axis and the dysregulation of the inhibitory feedback mechanism. Thus, the inflammatory response stimulates the HPA axis directly (through cytokine action) and indirectly (through glucocorticoid resistance) [150]. In major depressive disorders with melancholic features, a positive correlation has been found between the severity of the disease and concentrations of cortisol and IL-6 [151]. On the other hand, there is evidence that antidepressants normalize levels of proinflammatory cytokines and restore the feedback inhibition of the HPA axis [145,152]. In addition, an increase in proinflammatory cytokine concentrations may induce the tryptophan–kynurenine pathway and the generation of neurotoxic end products, as previously explained [150].

Oxidative stress and uncontrolled production of ROS play important roles in the hyperactivation of the immune pathways and the upregulation of cytokine production [77,153]. Enhanced production of proinflammatory cytokines and oxidative stress are tightly intertwined processes. Excessive production of ROS results in the oxidative damage of various cellular structures and the production of danger signals (DAMPs) that initiate inflammatory responses and microglial activation. Following activation, microglia, the resident cells of the innate immune system in the brain, produce proinflammatory cytokines and ROS. In a vicious loop, the ROS produced induce oxidative stress, supporting the ongoing microglial activation and further increasing ROS concentrations [133,154,155].

DAMPs-initiated signaling cascades transduce the signal to the nucleus and activate transcription factor nuclear factor κB (NF-κB). The activation of the NF-κB cascade triggers the production of proinflammatory cytokines, reactive oxygen and nitrogen species, and other potentially neurotoxic molecules such as inducible nitric oxide synthase (iNOS). Increased activation of iNOS results in increased production of NO, which, in excess, acts as a powerful oxidant. As mentioned previously, the superoxide anion along with the NO produced by iNOS form extremely dangerous peroxynitrite radicals. Thus, sustained oxidative injury, the neuroinflammatory response, and microglial activation are largely driven by the NF-κB pathway, whereas this pathway’s inhibition prevents the induction of the inhibitory effects of chronic stress on neurogenesis and the appearance of depressive behavior [156,157]. In animal models of neuroinflammation and depression, the upregulation of the NF-κB pathway has been demonstrated in the prefrontal cortex and the hippocampus [158,159]. However, in the human population, the results are not so consistent. For example, the activation of the NF-κB pathway was not observed in the peripheral blood mononuclear cells of adolescents and depressed medical students [160,161].

Several enzymes that are downstream targets of NF-κB may affect the course of depression. Such enzymes include cyclooxygenase 2 (COX-2) and NADPH oxidase. COX-2 inhibitors have been observed to have a positive effect on neuronal inflammation and the severity of the depression [162,163,164], whereas a single-nucleotide polymorphism of the *COX-2* gene may represent a risk for recurrent depressive disorder [165]. NADPH oxidase contributes to oxidative stress and neuroinflammation by overproducing the superoxide anion. As with COX-2, the pharmacological inhibition of NADPH oxidase presented beneficial antidepressant effects [166].

## 3. Experimental Approaches and Behavioral Tests for Studying Depressive-like Behavior in Animals

Although several animal models of depression are available, none of them address all the aspects of human depression. Chronic-unpredictable-mild-stress (CUMS)-induced depression is considered to be the most reliable model. The corresponding protocol implies the application of various stressors varying in duration and severity according to an unpredictable schedule for 2–3 weeks (typically). Such stressors may include forced swimming in cold or warm water, wet or soiled bedding, food and/or water deprivation, the inversion of the light/dark cycle, overnight illumination, cage tilting, cage shaking, the generation of noise, electric shocks applied to the feet, tail pinching, tail squeezing, 24 h social isolation, etc. CUMS results in the reorganization of cortical and limbic areas, which probably underlies depressive-like behavioral abnormalities [167,168,169]. There are also some other approaches that induce similar neurochemical, endocrine-related, and behavioral characteristics of human depression, such as olfactory bulbectomy, the social defeat model, the chronic restraint stress model, and the prolonged administration of glucocorticoids (reviewed in [170]).

The antidepressant effects of the pharmacological agents of interest can be further monitored using several behavioral tests (described in detail in [171,172,173]). The forced swimming test (FST) is one of the most applied tests for estimating the efficacy of a particular drug as an antidepressant agent. Animals are exposed to unescapable stress and forced to swim in a container filled with water. The total immobility period (wherein animals float motionless, keeping their heads above water) indicates the depressive state of the animal. Struggling (active movements to escape) and swimming can also be monitored. Modified versions of the test can also be used.

As false positive results may be obtained with drugs that increase spontaneous locomotor activity, the open field test (OFT) is often used to exclude the possibility of the stimulatory effect of a drug. In this case, animals are placed at the center of an arena whose floor surface is divided into squares. In the OFT, various parameters can be monitored to estimate the effect of a drug on locomotor behavior, including the number of line crossings, the time spent in the center of the field, the number of entries in the peripheral zone, and the time spent in the peripheral zone, together with the number of rearing activities, which is assessed to monitor anxiety [167,174,175].

The tail suspension test (TST) is used to estimate the antidepressant effect of a particular compound. In the TST, animals are suspended above the floor by taping their tails. The parameter that is measured is immobility time as an indicator of behavioral despair [169].

The sucrose preference test (SPT) is used as an indicator of anhedonia. Usually, two bottles of water are placed in different areas of the cage. After adaptation, water in one bottle is replaced with 10% sucrose so that the animal can choose between two drinks. The positions of the bottles are switched on a daily basis to reduce side bias [176].

Additional tests are very frequently used to estimate the cognitive status of the animals. The passive avoidance task evaluates memory acquisition. The apparatus used has light and dark chambers, wherein a wire grid is placed on the floor of the dark compartment that delivers a shock to the feet of the animal that enters. Short-term and long-term memory retention can be estimated using this task. The novel object recognition test is usually performed in a rectangular arena. Animals are placed in the middle of an open field containing two identical objects and allowed to familiarize themselves with the object and arena for several minutes. In the testing phase, the animals are returned to the arena, wherein one object has been replaced with a new one, which should cause the animals to demonstrate greater exploratory activity around the novel object. The parameter that is usually calculated is a discrimination ratio (the time spent exploring the novel object divided by the total exploring time) that estimates recognition memory [177].

As anxiety is often diagnosed alongside depression, in addition to the OFT, the elevated plus maze test is usually employed to assess the antianxiety potential of pharmacological agents. It uses a device—made of two open and two closed arms—that is elevated above the floor. The animals are placed in the central area with their heads directed toward a closed arm. The animals are allowed to explore the open and closed arms, usually for three minutes, and the overall result is represented as the total time spent exploring all arms [178].

## 4. Antidepressant Effects of Flavonols

Current therapeutic strategies for the treatment of depression mainly focus on balancing neurotransmitter levels, often with limited efficacy and many side effects. Hence, searching for novel, multitarget alternatives with more rapidly acting effects and better efficacy and tolerability is of the highest priority in the medical and scientific community. Considering the importance of oxidative and inflammatory mechanisms in the pathophysiology of depression, it has been suggested that compounds able to re-establish redox homeostasis and suppress inflammation might be useful for relieving depressive symptoms. Nowadays, there is a great deal of interest in the use of natural polyphenolic compounds as antidepressant agents, mainly due to their proven powerful antioxidative, anti-inflammatory, and neuroprotective effects [179,180,181,182]. These bioactive molecules, of which flavonoids are of particular interest, are abundantly present in fruits and vegetables and also available in the form of commercial dietary supplements.

Based on epidemiological data, diets rich in polyphenols have many health-beneficial effects. From the perspective of depression, besides demonstrating a plethora of antioxidative and anti-inflammatory effects, polyphenols from the diet may regulate the activity of the HPA axis and normalize levels of glucocorticoids, stimulate BDNF production and neurogenesis, inhibit the activity of MAO isoforms, restore neurotransmitter balance, mitigate clinical symptoms, and improve cognitive deficits [175,183,184].

As flavonoids represent a large and diverse family of natural polyphenolic compounds, we focused on flavonols due to their dietary abundance and promising antidepressant activity [185]. Hence, the second part of this review summarizes the mechanisms of action of various flavonols and evaluates their potential for use in adjuvant therapeutic approaches to attenuating symptoms of depression.

### 4.1. Antidepressant Effects of Quercetin

Quercetin (3,3′,4′,5,7-pentahydroxyflavone), the best-studied compound from this class, is one of the most ubiquitous dietary flavonoids. It can be found in many fruits, vegetables, and beverages, particularly in onions, apples, green tea, and various berries [186,187]. In diverse experimental settings, quercetin displayed anti-oxidative, anti-inflammatory, and neuroprotective effects [188,189]. In general, quercetin has demonstrated powerful antioxidative effects. It acts as a direct ROS scavenger and can increase the levels and activity of the endogenous mechanisms of antioxidative defense (both enzymatic and non-enzymatic) [190,191]. It also provided protection against H_2_O_2_-induced neuronal injury [192,193,194]. H_2_O_2_ is the most abundant endogenous ROS and is generated during oxidative deamination by MAO enzymes. Besides neutralizing the toxic effects of H_2_O_2_, quercetin also directly inhibits MAO-A [184,195]. The inhibitory effect of quercetin on MAO-B activity has also been shown in vitro [196].

Besides acting as an antioxidative agent, quercetin also displays prominent anti-inflammatory properties. In cultured microglial cells, it prevented microglial proliferation and its phagocytic activity, ROS production, and the activation of the NF-κB pathway; suppressed the activation of the NOD-, LRR-, and pyrin-domain-containing protein 3 (NLRP3) inflammasomes; promoted mitophagy (to alleviate mitochondrial oxidative stress) and mitochondrial function; and the expression of IL-1β [197]. The same study demonstrated the ability of quercetin to protect neuronal cells against lipopolysaccharide (LPS)-induced microglial activation and neurotoxicity in a mouse model of depression.

**Figure 2:**
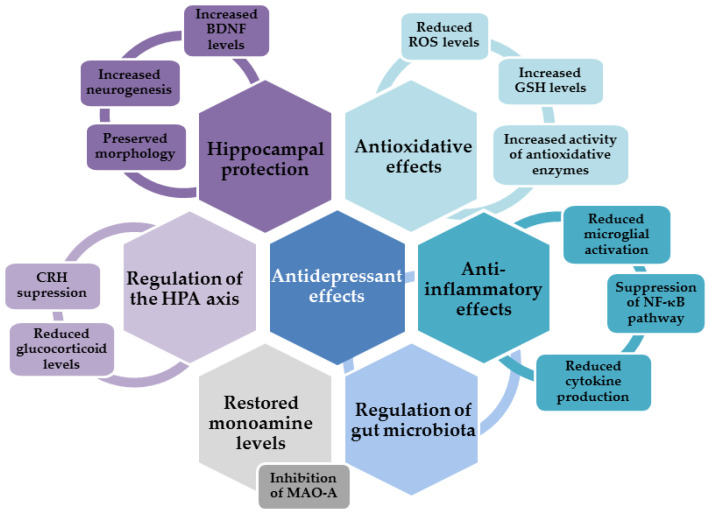
The main mechanisms contributing to antidepressant effects of quercetin and other flavonols.

Many behavioral tests performed using animal models have demonstrated beneficial effects of quercetin with respect to alleviating depressive-like behaviors (Figure 2, [198,199]). Several studies have shown anti-depressive effects of quercetin against CUMS-induced depression. Quercetin reversed CUMS-induced behavioral changes observed in the modified FST (wherein it increased swimming and escape attempts by climbing and decreased immobility time), TST (wherein it decreased immobility time), and OFT (wherein it increased number of field crossings and rearing behaviors). In the research in question, treatment with orally administered quercetin (25 mg/kg) started 2 weeks from the beginning of the CUMS protocol and lasted for 4 weeks, while the CUMS protocol was applied for 6 weeks. Behavioral improvements were accompanied by increased serotonin levels and improved oxidative stress parameters. Quercetin increased the activity of SOD and catalase, increased GSH content, and reduced glutamate levels, thus indicating the important role of the attenuation of oxidative stress and excitotoxicity in terms of its antidepressant effects. The CUMS-induced enhancement of TNF-α and IL-6 was also reversed by quercetin, further suggesting the contribution of anti-inflammatory mechanisms in the observed antidepressant effects of quercetin [169]. In a similar study (CUMS for 21 days and quercetin applied at a dose of 40 mg/kg), the antidepressant effect of quercetin was confirmed through improved results on the SPT and FST. At least partially, the stimulation of the hippocampal nuclear-factor-E2-related-factor-2 (Nrf2)-related signaling and inhibition of iNOS activity constituted the underlying mechanisms of this action [200]. Yet another study has shown that quercetin prevents behavioral abnormalities in animals exposed to chronic, unpredictable stress for 21 days. Quercetin, administered orally at a dose of 30 mg/kg during these 21 days, reduced anxiety (estimated using an elevated plus maze) and depressive-like behavior (SPT), improved memory retention (evaluated via a passive avoidance step-through task), and normalized locomotor activity (OFT) in stressed animals. Quercetin also attenuated oxidative stress in the hippocampus. It preserved total thiol content; reduced levels of thiobarbituric-acid-reactive substances (TBARS), which constitute a common measure of the severity of lipid peroxidation; reduced NO production; and improved catalase activity. Furthermore, at the gene level, it attenuated the expression of the proinflammatory markers IL-6, TNF-α, IL-1β, and COX-2 in the hippocampus. These antioxidative and anti-inflammatory effects had neuroprotective effects on hippocampal neurons, largely preventing their morphological changes and chronic stress-induced damage [167]. Besides regulating levels of serotonin and glutamate, it has been suggested that the inhibitory effects of quercetin on cholinergic nerves may contribute to this flavonol’s antidepressant activity [199].

Quercetin also suppressed an acute stress response induced by water immersion and restraint (WIR) stress in Wistar rats. Quercetin administration (50 mg/kg) reduced plasma levels of WIR-induced stress hormones ACTH and corticosterone and attenuated the expression of CRH mRNA in the hypothalamus. In addition, quercetin regulated the DNA-binding abilities of glucocorticoid receptors and cyclic adenosine 3′,5′-monophosphate (cAMP) response-element-binding protein (CREB). These transcription factors act as critical negative and positive regulators of transcriptional CRH expression, respectively. Kinases ERK1/2 are upstream of CREB, and quercetin suppressed the WIR-stress-induced phosphorylation of ERK1/2 as well. Altogether, these results imply that the role of quercetin in stress response control is mediated through the regulation of glucocorticoid receptors, CREB, and ERK1/2, resulting in the transcriptional suppression of CRH mRNA, which is the primary step in the activation of the HPA hormonal cascade [201]. Other studies also suggested that the antidepressant effects of quercetin are mediated through the suppression of CRF mRNA expression [202] and the ability to regulate the HPA axis [203]. However, in diabetic mice, the antidepressant effect of quercetin was determined to be independent of the HPA axis, as quercetin failed to modify levels of ACTH and corticosterone [204]. In another study performed using LPS-challenged rats, quercetin (40 mg/kg) increased the saccharin preference index and the immobility time in the FST, together with the normalization of synapsin-1, Copine 6, and BDNF levels, which might indicate that the antidepressant effect was mediated through the restoration of the animals’ BDNF levels and expression of synaptic plasticity-related proteins [205].

Mild depression-like behavior can also be induced by chemotherapeutic agents such as adriamycin. In animals exposed to adriamycin, quercetin (60 mg/kg), which was applied 72 h later, reversed adriamycin-induced behavioral changes in the FST (it reduced immobility, increased swimming, and prolonged struggling behavior in comparison with the adriamycin group), restored corticosterone levels, normalized the number of immune cells and improved brain oxidative status as evidenced by the increased GSH content, decreased the activity of glutathione-S-transferase, and reduced MDA levels [175]. The antidepressant effect of quercetin was further demonstrated in an olfactory bulbectomy model. The ablation of olfactory bulbs in rodents induces hyperactivity in the OFT and increases immobility time in the FST. In addition, it results in the hyperactivation of the HPA axis, an increase in corticosterone levels, the impairment of hippocampal neurogenesis, the promotion of oxidative and nitrosative stress, the upregulation of markers of neuroinflammation, and the stimulation of neuronal apoptosis in cerebral cortex and hippocampus [206]. Quercetin (40 and 80 mg/kg) applied together with the microglial inhibitor minocycline demonstrated protective effects and attenuated behavioral, molecular, and histopathological abnormalities induced by olfactory bulbectomy. It reduced MDA levels and nitrite accumulation (as an indicator of NO production), improved GSH content and SOD and catalase activity, prevented caspase-3 activation, restored serum corticosterone levels, and reduced the secretion of IL-6 and TNF-α. These antioxidative and anti-inflammatory effects of quercetin preserved the normal histology of the cerebral cortex and hippocampus, attenuated the proliferation of microglial cells, and shortened the immobility time of the bulbectomized animals in the FST, thereby demonstrating its antidepressant effects [206].

The effects of quercetin isolated from plants were also studied. Accordingly, it was observed that quercetin and quercetin 4′-O-glucoside isolated from the outer scale of an onion (*Allium cepa*) demonstrated antidepressant-like effects in mice when applied at doses of 10 and 20 mg/kg. The effects of quercetin and quercetin 4′-O-glucoside were studied using the FST and OFT. Both quercetin and quercetin 4′-O-glucoside significantly decreased the immobility time without displaying changes in the OFT, indicating their anti-depressive effects. However, as only the effects of quercetin 4′-O-glucoside were similar to those of fluoxetine, its effects were investigated further against CUMS-induced depressive-like behavior. Quercetin 4′-O-glucoside reversed CUMS-induced behavioral changes in locomotor activity and prevented a decrease in sucrose preference (a measure of anhedonia), thus improving the animals’ capacity to experience pleasure. Together with these behavioral changes, quercetin 4′-O-glucoside reduced MAO-A activity and increased serotonin levels. Furthermore, it restored GSH levels and the accumulation of TBARS, altogether suggesting that the antidepressant effects of quercetin 4′-O-glucoside were mediated by its antioxidative abilities and MAO-inhibitory property that restored serotonin levels [207].

Likewise, the consumption of food rich in quercetin also presented antidepressant-like effects. The antidepressant potential of onion powder was evaluated in a rat model of depression in the FST, but, in contrast to some previous findings, the effect was independent of the HPA axis’s regulation [208]. Hence, the levels of quercetin metabolites and range of dietary intake amounts needed for the attenuation of the HPA cascade should be addressed in future studies. The extract of *Ginkgo biloba* leaves, which is rich in quercetin glycosides, also reduced the immobility time in the FST and TST [209]. In addition, food enriched with quercetin (2 g/kg) was efficient in alleviating depressive behaviors in mice exposed to chronic social defeat stress. This was at least partially due to the reduced reactivation of astrocytes in the prefrontal cortex and hippocampus [210].

Hence, based on all these findings, the administration of quercetin can be considered a rational therapeutic approach to combat depression. It targets all of the main mechanisms contributing to the development of pathological processes, namely, oxidative stress, neuroinflammation, the dysfunction of the HPA axis, neurotransmitter levels, neuroplasticity, and neurogenesis. However, despite the convincing in vitro and preclinical evidence that suggests its remarkable potential with respect to alleviating depressive symptoms, there is still no adequate evidence from clinical trials to support its efficacy toward depressed patients. These studies are urgently needed to elucidate the exact capacity of quercetin as an antidepressant agent, to determine the optimal dose and the exact time frame for therapy, and to recognize eventual undesirable side effects when applied alone or in combination with standard therapy.

### 4.2. Antidepressant Effects of Myricetin and Myricitrin

Myricetin (3,3′,4′5,5′,7-hexahydroxylflavone) is a flavonol with a highly hydroxylated polyphenolic backbone. It is present in various vegetables, fruits, nuts, berries, and beverages [211]. As with quercetin, several studies have confirmed the neuroprotective effects of myricetin, which have largely been assigned to its antioxidative and anti-inflammatory properties [212]. For example, myricetin ameliorated cerebral-ischemia-induced-brain damage. Applied at a dose of 20 mg/kg, it reduced ROS levels and lipid peroxidation and promoted the activity of antioxidative enzymes SOD and catalase. These affordances improved mitochondrial function and stimulated the Nrf2 signaling pathway, resulting in reduced oxidative stress and the prevention of apoptotic death [213]. It has also been shown that myricetin may protect hippocampal neurons from damage [214]. However, caution is required, as in certain environmental conditions, such as increased concentrations of transient metal ions, myricetin may potentiate the production of ROS and exacerbate neuronal damage [215]. In this regard, the results of the meta-analysis have shown that depressed patients have increased blood levels of copper, which may exacerbate oxidative stress and systemic inflammation [216,217].

In mice exposed to repeated restraint stress for 21 days, myricetin (50 mg/kg) reversed the increase in the immobility time in the FST and TST without affecting locomotor activity in the OFT. Myricetin also reduced plasma ACTH and corticosterone levels, increased BDNF production, and partially restored the activity of glutathione peroxidase in hippocampal neurons [218,219]. In male rats exposed to a single prolonged stress (a model for the induction of post-traumatic stress disorder, which is also accompanied by depression), myricetin was applied for 14 days following stress induction. At a dose of 20 mg/kg, it reduced depression-like behavior in the FST and SPT, reduced the increase in plasma ACTH and corticosterone levels, normalized levels of serotonin and norepinephrine in the hippocampus and prefrontal cortex, and induced the hippocampal production of BDNF and its receptor, tropomyosin-related kinase B (TrkB), by activating the ERK signaling pathway [220]. In SAMP8 mice displaying a phenotype of accelerated aging, the intake of myricetin also induced the production of BDNF and nerve growth factor (NGF) by increasing CREB phosphorylation [221].

Similar effects, i.e., a reversal of depression-like behavior in the FST and TST, reduced corticosterone levels, and increased activity of hippocampal SOD and glutathione peroxidase in mice exposed to different forms of stress for seven consecutive days, have been observed following an administration of the crude ethanolic extract of *Saraca asoca* flowers, which contains high amounts of myricetin [222].

Myricitrin (myricetin-3-*O*-α-rhamnoside), the glycosylated form of myricetin, is a natural flavonol present in plants from the genera *Myrica*, *Eugenia*, and *Pouteria.* Its antioxidant and anti-inflammatory properties have been confirmed in several in vitro and in vivo studies [182,223]. Applied at a dose of 10 mg/kg for 21 days, myricitrin reduced the animals’ immobility time in the TST without modifying their general locomotor activity. It also promoted cell proliferation in the subventricular zone and dentate gyrus [224]. In a chronic mild stress model, myricitrin (at a dose of 10 mg/kg for 14 days) reversed depressive-like behavior, which was confirmed by an increased immobility time in the FST and TST, reduced adrenal hypertrophy as an indirect measure of the HPA axis’s activation, and attenuated IL-6 levels in the hippocampus [225].

### 4.3. Antidepressant Effects of Rutin

Rutin (quercetin-3-O-rutinoside) is a flavonol with a rutinose (a disaccharide consisting of rhamnose and glucose) attached to the quercetin aglycone. In animal studies, it demonstrated antioxidative, anti-inflammatory, neuroprotective, and antidepressant effects [226,227,228,229]. Thus, in a rat model of streptozotocin-induced neurotoxicity related to oxidative damage and inflammation, rutin, which was applied for 3 weeks at a dose of 25 mg/kg, attenuated lipid peroxidation, nitrite accumulation, and the reduction in the amount of GSH and stimulated the activity of glutathione peroxidase, glutathione reductase, and catalase in the hippocampus. Regarding its anti-inflammatory activity, rutin reduced microglial activation and expression of COX-2, iNOS, and NF-κB, altogether preserving hippocampal morphology and cognitive functions [230].

In Swiss albino mice, rutin administered for 16 days (30 mg/kg, 60 mg/kg, and 120 mg/kg) reduced the animals’ immobility time in an open-spaced FST and TST, indicating its anti-depressant potential. As the line-crossing in the OFT was increased, the authors suggested that the observed increase in locomotor activity reflected a rutin-induced increase in brain monoamine levels due to the inhibitory effect on MAO-A activity [231]. Similarly, in rats exposed to excess cadmium, rutin (50 mg/kg) induced protective effects by reducing the increase in cadmium-induced MAO activity and by increasing the activities of the antioxidant enzymes SOD and catalase in the hippocampus and cortex [232]. In a study that investigated the neurotoxic effects of silver nanoparticles, rutin reduced oxidative stress severity in the brain (it reduced MDA content, upregulated GSH, and increased activities of SOD, catalase, and glutathione peroxidase), normalized neurotransmitter levels (including the levels of serotonin, dopamine, and norepinephrine), regulated the transcriptional levels of MAO-A and MAO-B, improved histopathological changes induced by silver nanoparticles, and prevented astrogliosis and axonal demyelination [233]. The inhibitory effect of rutin on the MAO-B isoform’s activity has also been shown in in vitro studies [196,234]. Among several tested flavonoids, rutin displayed the most potent inhibitory activity. As all the other flavonoids had only one sugar molecule in their structure, it was suggested that sugar portions determine the strength of MAO-B inhibition. In another study, orally administered rutin decreased the immobility time in the TST at doses of 0.3, 1, and 3 mg/kg, demonstrating a positive effect on behavioral despair. This effect in the TST was prevented by a pretreatment with the inhibitors of serotonin and norepinephrine synthesis, suggesting that rutin increases the synaptic levels of these monoamines. On the other hand, rutin applied at these doses did not affect the number of crossings in the OFT, implying that its antidepressant effects were specific and not attributable to psychostimulatory activity [174]. The effects of rutin were also evaluated in mice exposed to CUMS for 21 days. Rutin improved behavioral deficits and induced antidepressant effects (it increased the preference for sucrose and pleasure), reduced anxiety (it enhanced the number of entries and the time spent in the center), and rescued cognitive functioning and motor coordination by preventing neuronal loss and preserving morphology in the CA3 region of the hippocampus [176]. In rats administered reserpine to induce depression (wherein a prolonged administration of reserpine induces monoamine depletion), rutin at a dose 80 mg/kg increased the number of crossings and rearing in the OFT and increased swimming and climbing behaviors in the FST, thus indicating the increased ability of the animals to cope with unavoidable stress conditions. A lack of helplessness was also confirmed via the TST [235].

Rutin also improved symptoms of depression by regulating the HPA axis. The oral administration of rutin (50 mg/kg) to animals exposed to CUMS improved levels of ACTH and corticosterone and normalized behavioral deteriorations in the OFT and FST. Rutin also increased the animals’ intake of sucrose solution in a fluid consumption test [168].

In addition to pure rutin, botanical extracts containing rutin as one of their major constituents also demonstrated antioxidant, anti-inflammatory, and anti-depressive effects; decreased serum corticosterone levels and attenuated the HPS axis; increased the expression of CREB and BDNF; and inhibited the activity of MAO-A [174,236,237].

### 4.4. Antidepressant Effects of Avicularin

Avicularin (quercetin-3-α-L-arabinofuranoside) is a flavonol isolated from several plants, including *Polygonum aviculare* [238]. The effects of fluoxetine and avicularin have been compared in a mouse model of depression induced by chronic exposure to unpredictable mild stressors. Avicularin (1.25, 2.5, or 5.0 mg/kg/d) demonstrated anti-inflammatory effects through the inhibition of the ERK/NF-κB pathway and the production of the proinflammatory cytokines IL-1β, IL-6, and TNF-α in the hippocampus. In addition, it inhibited the expression of iNOS, COX-2, and caspase-3. As the upregulated production of proinflammatory mediators plays an important role in the development of depression, the anti-inflammatory activity of avicularin was reflected in the improved results on behavioral tests (SPT, FST, and TST). At the highest dose, avicularin was as effective as fluoxetine in alleviating depressive-like behavior [239]. A prominent anti-inflammatory activity of avicularin mediated through the activation of the ERK signaling pathway was also observed in LPS-induced RAW 264.7 macrophage cells [240].

### 4.5. Antidepressant Effects of Fisetin

Fisetin (3,3’,4’,7-tetrahydroxyflavone) is a flavonol abundantly present in strawberries. Like other flavonols, fisetin may attenuate oxidative injury and neuroinflammation and mitigate neuronal damage. Many studies have demonstrated the powerful antioxidant ability of fisetin. Fisetin can reduce the severity of oxidative stress and the production of ROS and RNS, stimulate the activity of antioxidant enzymes and increase GSH levels, maintain redox balance and mitochondrial function, and prevent accompanying neuroinflammation. At the molecular level, fisetin modifies the activity of several signaling pathways involved in the determination of cell death or survival, including the PI3K/Akt, Nrf2, NF-κB, protein kinase C (PKC), and MAPK signaling pathways [241,242,243,244,245]. In a murine model of vascular dementia, the administration of fisetin at a dose of 40 mg/kg reduced lipid peroxidation and the death of hippocampal neurons, attenuated the activation of microglia and astrocytes, and promoted BDNF expression. In the same study, it reduced the number of apoptotic cells; upregulated the expression of the antiapoptotic protein Bcl-2 and downregulated expression of proapoptotic Bax; inhibited the inflammasome pathway; and reduced nuclear levels of NF-κB and the release of the pro-inflammatory cytokines IL-1β and IL-18, probably by preventing the ROS-mediated activation of the NF-κB/NLRP3 inflammasome and stimulating the antioxidative Nrf2/HO-1 pathway [246]. Similarly, regarding a brain injury induced by cerebral ischemia-reperfusion, fisetin reduced cell damage and the production of proinflammatory cytokines, inhibited iNOS and COX-2, and improved antioxidant parameters. These effects were mediated through the inhibition of the NF-κB pathway [247]. Likewise, the neuroprotective potential of fisetin against a spinal cord injury in rats was mediated by similar mechanisms. Fisetin (20 and 40 mg/kg) prevented a spinal-cord-injury-induced increase in proinflammatory markers (cytokines TNF-α, IL-1β and IL-6, iNOS, and COX-2) and the activation of the NF-κB/IκBα pathway and demonstrated antiapoptotic effects. It also upregulated the transcription of BDNF mRNA [248]. Hence, although these effects were not shown in animal models of depression, they clearly indicate that fisetin may modulate molecular and cellular processes involved in depression.

In mice, it has been shown that fisetin (10 and 20 mg/kg) may inhibit the immobility time in the TST and FST without affecting locomotor activity. Pre-treatment with p-chlorophenylalanine (PCPA), a selective inhibitor of tryptophane hydroxylase, nullified these beneficial effects. Furthermore, fisetin increased levels of serotonin and norepinephrine in the prefrontal cortex and hippocampus and inhibited MAO-A activity, suggesting that its antidepressant ability relies on its ability to normalize brain monoamine levels [249]. An inhibitory effect on MAO activity has also been demonstrated in astrocytes; this was an important finding, as the attenuation of glial MAO activity may aid neuronal protection [250]. In another study, a depressive-like behavior in mice was induced by LPS exposure. Pretreatment with fisetin (20–80 mg/kg for 7 days) reduced the immobility time in the FSF and TST and reversed an LPS-induced increase in inflammatory mediators, including IL-1β, IL-6, and TNF-α, in the prefrontal cortex and hippocampus. Moreover, at higher doses, fisetin suppressed the expression of iNOS mRNA and nitrite generation by modulating NF-κB, implying that the antidepressant effects of fisetin are largely determined by its anti-inflammatory ability [251]. In mice exposed to spatial restraint stress for 2 weeks, simultaneous treatment with fisetin (applied at a dose of 5 mg/kg) prevented an increase in the immobility time in the TST and FST [252]. In the same study, an antidepressant effect of fisetin was demonstrated in Abelson helper integration site-1 (Ahi1) knockout mice with a depressive phenotype. The effects of fisetin were likely mediated by the activation of the TrkB signaling pathway in the hippocampus [252]. Similarly, in mice exposed to a restraint test for 28 days, fisetin (15 mg/kg) improved behavioral deficits and attenuated the activation of the NF-κB cascade [253].

Finally, in a model of reserpine-induced fibromyalgia, which is characterized by reduced monoamine levels and depressive-like behavior, fisetin (10 and 25 mg/kg) reduced the immobility time in the FST and TST, prevented a monoamine decrease in the prefrontal cortex, and reversed reserpine-induced changes in oxidative and nitrosative stress markers (specifically the levels of ROS, GSH, MDA, SOD, and NO) [254].

### 4.6. Antidepressant Effects of Kaempferol

Kaempferol is a flavonol present in various foods (fruits and vegetables) and beverages and is among the most abundant flavonoids in the human diet [187]. Its neuroprotective effects have been demonstrated in previous studies. These effects were largely determined by its prominent antioxidative and anti-inflammatory action and modulation of distinct signaling cascades, including the NF-κB, p38, and Akt pathways [255]. Kaempferol’s action along these pathways also underlies its antidepressant effects. Thus, in mice exposed to chronic social defeat stress for 10 days, kaempferol (administered at doses of 10 and 20 mg/kg for 28 consecutive days after stress) displayed antidepressant effects, which were partly induced by the stimulation of the activation of Akt/β-catenin signaling in the prefrontal cortex [256]. The upregulation of Akt/β-catenin signaling was associated with antioxidant effects (as evidenced by the increased activity of the antioxidant enzymes SOD, catalase, glutathione peroxidase, and glutathione S-transferase; reduced MDA content; and attenuated protein carbonylation) and anti-inflammatory activity (which was demonstrated through the attenuated release of the proinflammatory mediators IL-1β and TNF-α and microglial activation). Ultimately, these kaempferol-induced molecular changes contributed to the improved performance observed in behavioral tests. In particular, kaempferol increased sucrose consumption, time spent engaging in social interactions, and mobility time in the TST [256]. The administration of LY294002, a PI3K inhibitor, prevented improvements in behavioral deficits and the normalization of oxidative-stress- and neuroinflammation-related parameters, suggesting the important contribution of the Akt/β-catenin pathway in the anti-depressive action of kaempferol. In another study, kaempferol was administered at doses of 10, 20, and 40 µg/rat for 4 weeks. Depending on the dose, it improved performance in the TST and FST and suppressed MAO-A activity [257].

Antidepressant effects of kaempferol have also been observed in mice chronically exposed to restraint stress (2 h/day for 14 days). After being restrained, the mice were fed with a diet supplemented with kaempferol (30 mg/kg/day) for 14 days. Kaempferol reduced the immobility time in the TST and FST to the control level. In a rotarod test, the time of performance was also comparable to the control group. Moreover, kaempferol increased the plasma level of β endorphins, which may be relevant since opioid system has been implicated in the effects of certain antidepressants [258].

Yet another interesting study demonstrated that the supplementation with kaempferol-3-*O*-glucoside in female rats on an obesogenic diet during pregnancy and lactation may revert depression-like behavior in the female offspring [259].

A recent study demonstrated that sirtuin 3, the main mitochondrial deacetylase, had a prominent role in mediating the antidepressant and anxiolytic effects of kaempferol in an animal model of menopausal depression. The authors used two different approaches: one in which ovarian failure was induced by the ovotoxin 4-vinylcyclohexene diepoxide, and the other in which aged mice were exposed to CUMS for 8 weeks. Kaempferol (10 mg/kg for 14 days) improved performance in the behavioral tests (FST, OFT, and elevated plus maze) and increased the expression of sirtuin3 and SOD2 deacetylation (note that only the deacetylated form of SOD2 acts as an ROS scavenger), possibly by promoting the nuclear translocation of estrogen receptor α, which might increase the expression of sirtuin 3. Kaempferol also alleviated mitochondrial dysfunction, increased activity of mitochondrial SOD, and attenuated oxidative stress in the hippocampus. These findings confirmed the important contribution of oxidative stress in menopausal depression and the potential of kaempferol as an antidepressant agent [260].

Similar to quercetin and other flavonols, the lack of clinical data is the major obstacle for evaluating the potential of kaempferol as an antidepressant agent. It has been shown that the antioxidative and anti-inflammatory effects of kaempferol depend on the glycosylation pattern [255]. Hence, clinical studies with kaempferol and its glycosylated derivatives are urgently needed to address their potential as antidepressant agents among depressed patients.

### 4.7. Antidepressant Effects of Morin

Morin (2′,3,4′,5,7-pentahydroxyflavone) is a flavonol that was originally isolated from plants of the *Moraceae* family, although it is also abundantly present in many fruits, herbs, green tea, and red wine. It possesses prominent antioxidative and anti-inflammatory abilities [261,262], and may prevent acute stress-induced biochemical and behavioral changes [263]. Hence, as with other flavonols, its protective potential against depressive-like behavior has been recognized. In rats exposed to CUMS for 4 weeks, morin administered concomitantly with stressors (15 and 30 mg/kg) induced antidepressant effects. It improved results in the FST (increased swimming score and reduced immobility time), SPT (increased intake of sucrose), and OFT. Furthermore, in the cerebral cortex and hippocampus, it mitigated neurochemical and biochemical changes induced by unpredictable stressors. It increased monoamine levels (serotonin, epinephrine, and norepinephrine) and improved oxidative stress status (evidenced by decreased MDA levels and increased glutathione content). The results also indicated its possible role in inflammasome activation, as morin reduced levels of the inflammasome pathway markers TNF-α, IL-1β, toll-like receptor-4 (TLR-4), NLRP3, and caspase-1, indicating its promising potential in alleviating the inflammatory basis of the disease [264]. Another study performed on mice suggested that the antidepressant effects of morin could be mediated through the L-arginine-NO pathway, as beneficial effects of morin on despair-like behavior were reversed by the NO precursor L-arginine [265].

The neuroprotective effect of morin in a rat model of attention deficit/hyperactivity disorder (ADHD) has also been demonstrated. The study indicated a crucial role of antioxidative and anti-inflammatory mechanisms in attenuating the severity of the disease. Thus, morin improved oxidative stress parameters (by targeting Nrf2/HO-1 pathway), monoamine levels, and inflammatory status (by hindering the TLR4/NLRP3 pathway) in pups with ADHD [266].

### 4.8. Antidepressant Effects of Other Flavonols

Isorhamnetin, a 3′-O-methylated derivative of quercetin, is one of the major bioactive compounds that can be isolated from *Ginkgo biloba* L. leaves. Antidepressant effects of *Ginkgo biloba* L. leaves have been demonstrated in various behavioral tests in rodents [209,267]. As for other flavonols, the antioxidative and anti-inflammatory effects of isorhamnetin have been reported in different pathological conditions and model systems and were mediated through the PI3K/Akt, MAPK, Nrf2, and NF-κB pathways [268,269]. However, further studies are needed, as so far, no antidepressant effects of isorhamnetin have been shown.

Rhamnazin is a methylated derivate of quercetin with antioxidative and anti-inflammatory properties [270]. Chronic administration of rhamnazin (50 mg/kg) improved cognitive dysfunctions induced by chronic restraint in rats (4 h/day for 30 days, without access to food and water), decreased ACTH levels in the plasma, and restored hippocampal BDNF levels [271].

Gossypetin is a flavonol that is abundantly present in *Hibiscus* species. At a dose of 20 mg/kg, gossypetin demonstrated significant antidepressant activity in rats exposed to forced swimming [272].

Galangin is a flavonol whose antidepressant potential has scarcely been investigated. However, it enhanced the antidepressant effect of fluoxetine in a rat model of depression, indicating the possibility of using galangin in combination with SSRIs to improve clinical outcomes [273].

Isoquercetin (quercetin 3′-O-rhamnoside) is a quercetin glycoside with an attached glucose. Like quercetin, it is effective in reducing oxidative stress and levels of proinflammatory cytokines by inhibiting the Nrf2 and NF-κB pathways [274]. For isoquercitrin (quercetin 4′-O-rhamnoside), inhibitory MAO-B activity has been shown [196]. Its administration (at a dose of 0.6 mg/kg/day) may reduce levels of ACTH and corticosterone when given for 2 weeks [275].

Hyperoside (quercetin 3-galactoside; flavonol glycoside) is one of the major bioactive compounds from *Hypericum perforatum* that displays prominent antidepressant effects. Hyperoside demonstrated antidepressant activity in CUMS-induced mice through increased BDNF levels and reduced expression of the NLRP1 inflammasome. Behavioral effects were confirmed via the SPT, TST, and FST [276]. The anti-immobility effect of hyperoside in rats (10 and 20 mg/kg) was prevented by a D2 antagonist, suggesting that the dopaminergic system could be involved in the antidepressant effect of hyperoside [277]. Moreover, treatment with hyperoside (0.6 mg/kg/day) for 2 weeks reduced plasma levels of ACTH and corticosterone [275].

## 5. Flavonols and Gut–Brain Axis

Gut microbiota constitute a wide spectrum of commensal microorganisms (bacteria, fungi, and viruses) that regulate the metabolic, endocrine, and immune functions of a host [278]. A growing body of data suggests that the dysbiosis of gut flora plays a contributory role in the pathogenesis of various neuropsychiatric diseases, including major depressive disorder. Depression affects the composition of gut microbiota [279,280], and various depressants exhibit antimicrobial activity [281,282]. Moreover, rats that received fecal microbiota from depressed patients developed depression-like behavior, indicating the causative role of gut microbiota in neurobehavioral symptoms. Likewise, behavioral alterations were accompanied by reduced levels of hippocampal neurotransmitters and serum cytokines, which also supports the existence of a functional interplay between gut microorganisms and the pathogenesis of depression [283]. Communication between the gut microbiota and the brain, termed the gut–brain axis, is bidirectional. The brain may affect the composition and function of gut microbiota by producing cytokines and antimicrobial peptides, while, through the enteric nervous system and the vagus nerve, bacteria in the gut may transmit signal to the brain and regulate its functioning. Gut microbiota produce various neuroactive chemicals, including neurotransmitters (such as GABA, catecholamines, histamine, and acetylcholine), as well as their precursors which may reach the brain through blood circulation and modify the synthesis and concentrations of neurotransmitters in the brain [284].Besides, some bacterial metabolites may regulate the production of neurotransmitters by enteroendocrine cells [285]. Moreover, various inflammatory molecules originating from the gut microflora (lipopolysaccharides, endotoxins, etc.) may activate the peripheral immune system, reach the brain, activate microglia, and promote the development of depression through cytokine release [284,286,287]. The microbiota–inflammasome hypothesis of major depressive disorder states that the dysbiosis of the gut microbiota deteriorates the integrity of the blood–brain barrier through peripheral inflammation. This results in the inflammasome’s activation, the development of depressive symptoms, and further modulation of the gut microbiota [288,289]. On the other hand, intake of specific probiotics could contribute to the restoration of the health-promoting composition of the gut microbiota, prevent infiltration of the peripheral immune cells and microglial activation in the brain, and ultimately exert antidepressant effects [289]. It has been shown that many fecal metabolites and microbial genes distributed differently in patients and healthy individuals are related to amino acid metabolism (γ-aminobutyrate, phenylalanine, and tryptophan) and inflammatory imbalance [290]. Analysis of fecal samples also revealed that depressed patients have increased levels of *Bacteroidetes, Proteobacteria*, and *Actinobacteria* but a reduced relative abundance of *Firmicutes* [279]. Another study demonstrated that an abundance of GABA-producing *Bacteroides* negatively correlates with the functional connectivity in brain areas associated with depression [291]. Reduced abundance of *Bifidobacterium* and *Lactobacillus* is also more common in depressed patients [292]. Similarly, in a rat model of ACTH-induced depression, microbial profiling revealed a reduced number of *Lactobacillus* and *Akkermansia* together with an increase in *Ruminococcus* and *Klebsiella* [280].

Among other contributing factors, the composition of the gut microflora is influenced by diet. In this regard, dietary polyphenols can modulate the intestinal microenvironment and functions of gut microbiota, which may affect the outcome of a therapeutic treatment [286,293,294]. On the other hand, polyphenols, including flavonols, need to be metabolized and activated by microorganisms in the gut. Via microbial metabolism, dietary polyphenols are broken down into small aromatic metabolites, some of which may directly act as neurotransmitters [287,293]. In fact, interactions between the microbiota and polyphenols in the diet are important factors in determining the effects of polyphenols on brain functions and the biological hallmarks of depression. There is increasing evidence that gut microbiota and dietary polyphenols act in concert in the alleviation of symptoms of depression. In particular, polyphenols from the diet or herbal medicinal products modulate the composition of gut microbiota by directly promoting the growth of health-beneficial bacterial species, whereas gut microbiota produce numerous metabolites from dietary polyphenols, mainly in the form of bioactive phenolic acids, with antidepressant properties usually superior in comparison with standard therapies [295,296]. Thus, during biotransformation by gut microbiota, polyphenols and their intermediary metabolites are degraded into various phenolic acids and their derivatives (e.g., ferulic acid, caffeic acid, ellagic acid, chlorogenic acid, benzoic acid, and hippuric acid). These bioactive metabolites are produced through the specific enzymatic activity of bacterial species in the gut. As mentioned, these metabolites usually show better absorption and biological effects. Several studies have demonstrated that they may attenuate depressive-like behavior by regulating monoamine levels, the HPA axis, antioxidative defense, and inflammation [295,297].

In light of these findings, attempts have been made to evaluate the antidepressant potential of flavonols from the perspective of the regulation of gut microbiota [287]. Thus, in LPS-challenged laying hens and broiler chickens, quercetin ameliorated intestinal mucosal injury and inflammation, maintained intestinal functions, and modified microbial communities [298,299]. Some of these changes were related to the increased abundance of bacteria capable of producing short-chain fatty acids (SCFAs) (e.g., acetate, propionate, and butyrate) from dietary fibers. SCFAs have an important role in the regulation of neuro-immunoendocrine functions and the improvement of cognitive abilities [298,300]. Moreover, SCFAs may signal entero-chrommafin cells to increase the production of serotonin, which is the key signaling molecule of the gut–brain axis [297]. SCFAs may also increase the production of the hormone leptin, which was able to reverse CUMS-induced hedonic deficits in the SPT and behavioral despair in the FST, and increase neuronal activity in the limbic system, including the hippocampus, all of which may contribute to the antidepressant effects of polyphenolic compounds [297,301]. Treatment with quercetin also increased *Lactobacillus* counts [299,302]. Of note, intervention with *Lactobaccilus casie* modified gut microbiota and improved depression-like behavior in rats exposed to CUMS [303]. Quercetin and its derivatives are also highly metabolized by the gut microbiota; however, the biological effects of their major metabolites have yet to be investigated [304].

Thus, although the mentioned findings could be considered to be indicative and promising, further studies are needed to clarify if flavonols can exert antidepressant effects by improving intestinal homeostasis.

## 6. Conclusions

Following COVID-19, there has been a huge outbreak of major depressive disorder worldwide. Unfortunately, the treatment options for depression are still not satisfactory. Apart from side effects and withdrawal symptoms, many patients do not respond to therapy, and therapy yields disappointing improvements far too often. Pharmacological interventions are mainly focused on balancing solely monoaminergic neurotransmission. However, multitarget approaches offer many advantages compared to classical prescriptions. Bioactive compounds originating from plants constitute an option with fewer unwanted side effects and with remarkable multimodal activities. They show prominent antioxidative activity, anti-inflammatory and neuroprotective effects, and the ability to regulate the HPA axis, protect neurons in the hippocampus, and stimulate neurogenesis.

Results from recent studies indicate the targeting of oxidative stress and inflammation as a promising therapeutic option for alleviating symptoms of depression as both are critical events in the cellular and molecular pathogenesis of this disease. Polyphenols from the flavonol class of flavonoids exert prominent antioxidative and anti-inflammatory effects, and in various animal models of depression, they have demonstrated remarkable anti-depressant activity. Although many research efforts have been directed to the most abundant flavonols in the diet, such as quercetin and kaempferol, studies on other flavonols are accumulating, which generally support the notion of flavonols acting as potential antidepressant agents.

However, clinical studies have yet to investigate the full potential of flavonols in depressed patients. These studies should be correlated not only with monoamine levels but also with the markers of oxidative stress and inflammation and levels of the HPA axis hormones. Regarding serotonin rebalancing, it is also necessary to work intensively on better understanding the interplay between flavonols and specific types of serotonin receptors. Monitoring all these parameters will give better insights into flavonols’ capacity to alleviate molecular and cellular events in the brain and immune and endocrine systems that underlie the clinical symptoms of depression. As flavonols are relatively abundant in various fruits and vegetables, their incorporation in a daily diet, such as the Mediterranean one, which, among other beneficial effects, may also have a positive effect on the composition of gut microbiota, could be an effective approach for the maintenance of mental health and the prevention of or therapy for major depressive disorder. In general, the use of symbiotics (a combination of probiotics and dietary polyphenols) has been identified as a promising therapeutic approach. Due to the high variability of the composition of gut microbiota, it is likely that the simultaneous delivery of both probiotics and flavonols would be the best strategy for maximizing and standardizing the health-beneficial outcomes of flavonol administration. Symbiotics show great potential by targeting several pathological mechanisms, mainly those related to the attenuation of oxidative stress and neuroinflammation. However, as inter-individual differences related to genetic background, epigenetic modifications, lifestyle, and diet shape the interactions between gut microbiota and flavonols as well as their biotransformation into various metabolites in the lower colon, further studies are needed to optimize symbiotic formulas and better reveal the potential of flavonols against molecular and behavioral signs of depression. Finally, it should be emphasized that polyphenols from various groups, each with distinct biological effects, are simultaneously present in various plants, and their synergistic action potentially could exert even more prominent antidepressant action in comparison to flavonols alone, which needs to be investigated in further studies.

## Data Availability

Not applicable.
